# Association between social vulnerability index and cataract surgery care in medicare beneficiaries: a retrospective cohort study

**DOI:** 10.1186/s12886-026-05110-2

**Published:** 2026-07-09

**Authors:** Kelly C. Nguyen, Taylor Hall, Siqi Gan, Erica Langnas, John Boscardin, Catherine Q. Sun, Sei Lee, Catherine L. Chen

**Affiliations:** 1 University of California, San Francisco School of Medicine, San Francisco, CA USA; 2 Department of Anesthesia, Stanford University, Stanford, CA USA; 3 Division of Geriatrics, University of California, San Francisco, San Francisco, CA USA; 4 Northern California Institute for Research and Education, San Francisco, CA USA; 5 Philip R. Lee Institute for Health Policy Studies, University of California, San Francisco, CA USA; 6 Department of Anesthesiology and Perioperative Care, University of California, San Francisco, CA USA; 7 Department of Epidemiology and Biostatistics, University of California, San Francisco, CA USA; 8 Department of Ophthalmology, University of California, San Francisco, CA USA

**Keywords:** Social vulnerability index, Cataract diagnosis, Cataract surgery, Social determinants of health, Health disparities, Medicare

## Abstract

**Background:**

Social vulnerability index (SVI) has been used as a surrogate measure to assess social determinants of health (SDOH) when conducting health disparities research, but the impact of SVI on the timing, receipt, and outcomes of cataract care in a nationally representative population of patients is unknown.

**Methods:**

A retrospective observational cohort study of Medicare fee-for-service beneficiaries aged 66 + in 2014 without a prior diagnosis of cataract disease was conducted to determine who went on to develop cataract disease and undergo cataract surgery between 2014 and 2021. A Fine and Gray sub-distribution hazard model was used to determine the association between SVI quartile, time to diagnosis and time to cataract surgery. Logistic regression was used to assess the association between SVI quartile and the incidence of ophthalmic complications.

**Results:**

Among 170,778 Medicare beneficiaries aged 66 + years of age at the start of the study period, 50.2% identified as female, 7.0% Black, 4.4% Hispanic, and 80.9% White. Fewer beneficiaries residing in high SVI (higher vulnerability) regions were diagnosed with cataracts (Quartile 4 55.9% vs. Q1 67.1%, *p* < 0.001) and underwent cataract surgery (Q4 19.4% vs. Q1 21.1%, *p* < 0.001) compared to beneficiaries in low SVI regions. After adjusting for patient demographics, overall health and frailty status, and health systemic characteristics including region, urban/rural location and number of ophthalmologists per capita, patients residing in higher SVI areas had lower rates of diagnosis (Q2 HR: 0.92 95% CI (0.91, 0.94); Q3 0.86 (0.84, 0.87); Q4: 0.78 (0.77, 0.80), reference=Q1) and cataract surgery (Q3 HR: 0.95, 95% CI (0.92, 0.98) and Q4: 0.90 (0.87, 0.93), respectively, reference=Q1). However, once a diagnosis was made, patients residing in higher SVI regions had higher rates of cataract surgery (Q2 HR: 1.04 (1.01, 1.07); Q3: 1.06 (1.03, 1.09); and Q4: 1.08 (1.05, 1.12), respectively, reference=Q1). There was no association between SVI and ophthalmic complications.

**Conclusions:**

Higher SVI is associated with disparities in receipt of cataract surgery among Medicare beneficiaries, which may be mediated by delays in cataract diagnosis. Reducing SDOH-related barriers to timely diagnosis in under-resourced areas could shorten the overall time to cataract surgery in this population.

**Supplementary Information:**

The online version contains supplementary material available at 10.1186/s12886-026-05110-2.

Cataract surgery has been performed safely among older patient populations, and delays in treatment may result in increased morbidity and worse quality of life (QoL) in patients with cataract disease [1–6]. Researchers have shown inequities in the timing of cataract surgery, with individuals from certain racial, ethnic, or socioeconomic backgrounds experiencing longer wait times for surgery compared to others [7–9]. These delays can increase the risk of falls, motor vehicle accidents, depression, frailty, and vision loss [10–15]. The 2020 Centers for Disease Control (CDC) and Prevention/Agency for Toxic Substances and Disease Registry’s Social Vulnerability Index (CDC/ATSDR SVI) is a community marker for social vulnerability that has been used as a surrogate measure to assess social determinants of health (SDOH) when conducting health disparities research [16]. Prior studies have demonstrated that individuals from areas with high SVI scores experience delays in common surgeries, increased health care spending, and poorer surgical outcomes [17–20]. 

More recently, researchers have also noted disparities in ophthalmology appointment attendance when patients are stratified by their SVI percentile [21]. However, this study sample was limited to a single center and only looked at clinic visits rather than the spectrum of cataract surgery care. An unrelated single-center study found that racial minorities are more likely to experience both referral and procedural delays in cataract care [8], while other researchers found a higher rate of complex cataract surgery among non-White patients [9]. There have not been any studies that assess the association between patients’ SVI percentile and the receipt of cataract surgery in a nationally representative population of patients who have not yet been diagnosed with cataract disease.

To improve our understanding of SDOH-related disparities on cataract surgery care, using Medicare claims spanning from 2013 to 2021, we identified Medicare beneficiaries who were 66 years old at the time of enrollment and did not have a cataract diagnosis in or prior to 2014. We hypothesized that patients residing in higher SVI areas would experience more delays in cataract care and an increased incidence of perioperative complications compared to patients in low SVI areas. After stratifying our cohort into SVI quartiles, we assessed (1) the association between SVI quartile and the likelihood of receiving a cataract diagnosis, undergoing ocular biometry or having cataract surgery; and (2) the association between SVI and the incidence of perioperative ophthalmic complications. Ultimately, the aim of our study was to assess the impact of SVI on the timing, receipt, and outcomes of cataract care.

## Methods

### Study design

This was a retrospective cohort study evaluating the relationship between SVI, receipt of cataract surgery, and surgical outcomes.

### Data source

We obtained US Centers for Medicare & Medicaid (CMS) research identifiable files from 2013 to 2021 representing a 20% sample of Medicare beneficiaries. For each beneficiary, we accessed the Outpatient, Carrier, MedPAR, Master Beneficiary Summary File (MBSF) and MBSF Chronic Conditions Segment files. The last file originates from the CMS Chronic Conditions Data Warehouse (CCW), a research database created by the Centers for Medicare & Medicaid Services to link patient-level Medicare beneficiary, claims, and assessment data across the continuum of care [22]; CCW Condition Flags use claims-based algorithms to identify the first recorded date that beneficiaries satisfy the ICD-9 or ICD-10 claim criteria for a particular condition. Beneficiaries were followed longitudinally using encrypted CCW Beneficiary IDs, allowing for de-identified linkage across years. The CDC/ATSDR SVI was obtained from the CDC website [16]. This study was approved by the Institutional Review Board at the University of California, San Francisco (#21-35606) and was conducted in accordance with the Declaration of Helsinki.

### Study cohort

We identified patients who were 66 years of age in 2014 and enrolled in the Medicare fee-for-service program without a concurrent health maintenance organization (HMO) plan. HMO is a type of insurance plan in the US that requires patients to coordinate care and obtain specialist referrals through a primary care doctor and receive care from within the HMO network for insurance coverage. Patients less than 66 years old were excluded to have a 12-month look-back to assess comorbidities. We also excluded beneficiaries who (1) had a CCW flag for ever having been diagnosed with cataract disease, or Current Procedural Terminology or International Statistical Classification of Diseases, Ninth Revision (ICD-9) codes related to cataract disease or treatment on or prior to their birthday in the year 2014 (eTable 1); (2) could not be linked to a hospital referral region; (3) had cataract surgery performed emergently (eFigure 1).

### Cataract care events

A new cataract diagnosis was defined by the first date of an ICD-9 or ICD-10 code for cataract diagnosis or the first date that a CCW flag for cataract diagnosis appeared for that beneficiary after study enrollment (eTable 1). Completion of ocular biometry was determined using the *Current Procedural Terminology* (CPT) codes (76516, 76519 or 92136). Completion of cataract surgery was defined using the outpatient and carrier files and *CPT* codes for cataract surgery (66982, 66983, 66984). The interval between initial enrollment in the study cohort (2014) and events of interest was recorded in months. All patients were followed through either December 31, 2021, the date of first-eye cataract surgery, or the date of death, whichever came first.

### Definition of social vulnerability index

Census data was used to develop the CDC/ATSDR SVI to help identify communities that need support before, during, or after disasters [23]. The SVI is composed of sixteen US census variables from the 5-year American Community Survey (ACS) clustered into the following areas of social vulnerability (socioeconomic status, household characteristics, racial/ethnic minority status, and housing type and transportation).

For this study, each beneficiary’s 5-digit zip code from the MBSF files was linked to its corresponding SVI census tract using a weighted average of the SVIs of all the census tracts within each ZIP code. The weights used were the residential ratios from the HUD Zip-to-Tract Cross Walk [24, 25]. Patients were then stratified by ordinal quartiles according to their assigned SVI, with the lowest quartiles designated as lowest vulnerability and highest quartiles designated as highest vulnerability. The range of Social Vulnerability Scores is 0 to 1.

### Identification of perioperative complications

We used ICD-10 codes to identify ophthalmic complications within 30 days after each procedure (eTable 2) [26]. 

### Patient and health system characteristics

We identified additional covariates including patient characteristics such as age, gender, race/ethnicity, and health status (assessed using the previously validated Charlson Comorbidity Index [27] and the Kim frailty index [28]) as well as health system characteristics including geographic region [29] and the number of ophthalmologists/100,000 Medicare enrollee per hospital referral region (HRR) obtained from the Dartmouth Atlas of Healthcare [30]. Race and ethnicity were obtained directly from the Master Beneficiary Summary files, which is populated from Social Security Administration data on each beneficiary’s self-reported race and/or ethnicity [31]. We included race as a covariate in the study because others have reported racial disparities in cataract care [7, 8, 32]. Surgical volume was calculated at the patient level by assessing the total number of cataract surgeries their ophthalmologist performed within the same calendar year as the patient’s date of cataract surgery.

### Statistical approaches

In this study, the primary independent variable was the patient’s SVI. We conducted a descriptive analysis demonstrating patient baseline characteristics stratified by SVI and highlighted the relationship between SVI and race at each stage of cataract care. Continuous variables were compared using ANOVA and categorical variables were compared using the chi-square test. We also assessed standardized mean differences across continuous and categorical variables. We created cumulative incidence curves to illustrate how SVI affects the receipt and timing of cataract diagnosis and surgery. To determine the association between SVI quartile, time to diagnosis, time to biometry and time to cataract surgery, we created a series of Fine and Gray sub-distribution hazard models (which accounts for death as a competing risk), adjusting for patient demographics (age, sex, race/ethnicity), CCI and frailty index, geographic region and number of ophthalmologists per 100,000 population per HRR. While age was not included as a covariate in the models that included all beneficiaries (since all patients were age 66 at the time of study enrollment), among the subset of beneficiaries who received a cataract diagnosis, age at the time of diagnosis was included as a covariate in the model assessing time from diagnosis to cataract surgery. Multicollinearity was checked using variance inflation factors (VIF) (eTable 3), while the interaction between SVI and race was tested by adding an interaction term of SVI x race to all the models. However, we found no interaction between SVI and race in the hazard rate of cataract diagnoses, ocular biometry, or cataract surgery; therefore, the interaction term was not included in the final models.

We also created a mixed effects logistic regression model (with ophthalmologist as a random effect to account for clustering of patients at the level of the ophthalmologist) to assess the association between SVI quartile and the incidence of ophthalmic complications after accounting for the same covariates and surgical volume in the subset of patients who underwent cataract surgery and had at least 30-day follow-up to detect complications.

## Results

**Fig. 1:**
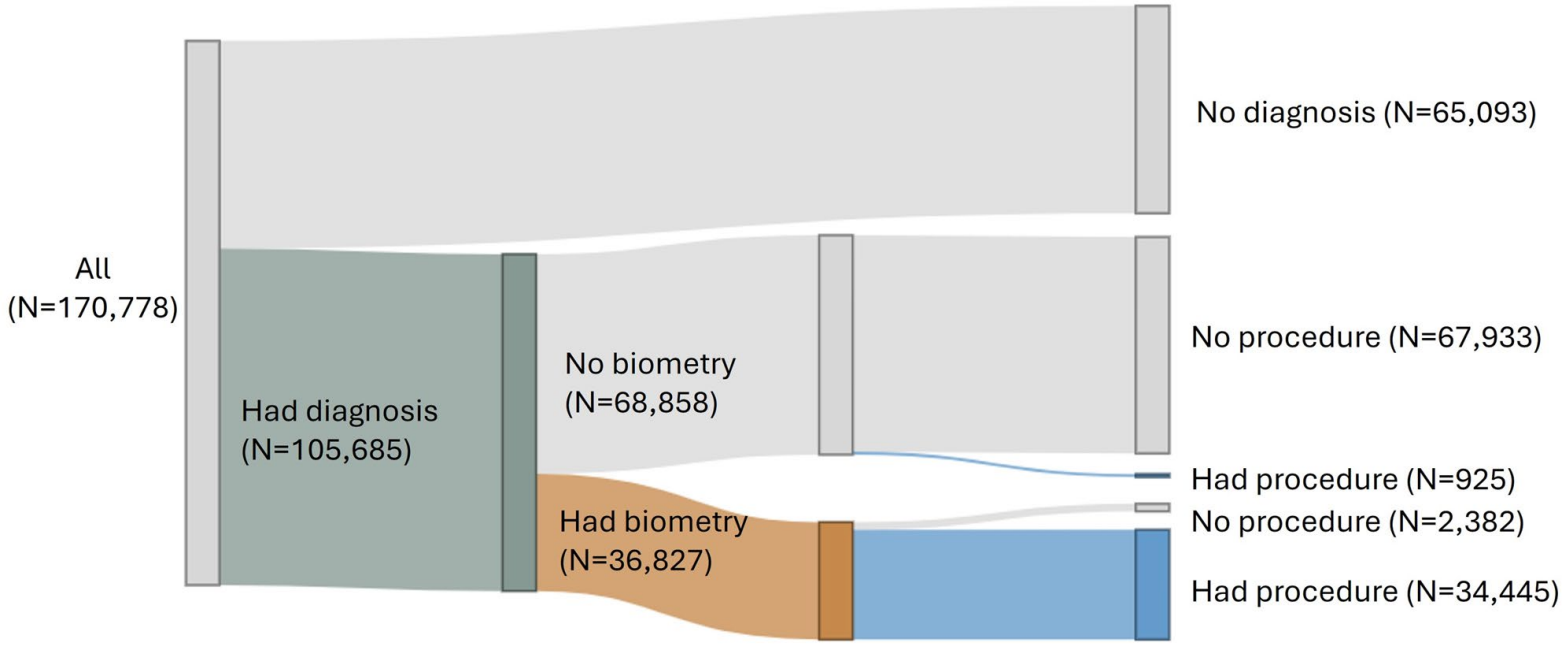
Proportion of cohort who had cataract diagnosis, ocular biometry, and cataract surgery procedure, 2014–2021

All 170,778 beneficiaries who met inclusion criteria were 66 years of age at the start of the study period; 50.2% identified as female, 7.0% Black, 4.4% Hispanic, and 80.9% White (Table 1). Among our study cohort, 61.9% had a cataract diagnosis (Fig. 1). Approximately 21.6% completed biometry and 20.7% had a cataract procedure. Among patients diagnosed with cataract, 34.8% had biometry; and 93.5% of the patients who had biometry went on to have cataract surgery.

**Table 1:** Baseline characteristics of medicare beneficiaries without cataract diagnosis at start of study

	SVI quartile				
	(lowest) Q1 (0.00–0.30)	Q2 (0.30–0.46)	Q3 (0.46–0.62)	(highest) Q4 (0.62–1.00)	Total
	*N* = 42,813	*N* = 42,757	*N* = 42,659	*N* = 42,549	*N* = 170,778
Patient					
Age	66	66	66	66	66
Gender					
Male	20,818 (48.6%)	20,983 (49.1%)	21,569 (50.6%)	21,626 (50.8%)	84,996 (49.8%)
Female	21,995 (51.4%)	21,774 (50.9%)	21,090 (49.4%)	20,923 (49.2%)	85,782 (50.2%)
Race					
Asian/Pacific Islander	875 (2.0%)	828 (1.9%)	855 (2.0%)	1,109 (2.6%)	3,667 (2.1%)
Black	1,008 (2.4%)	1,783 (4.2%)	2,596 (6.1%)	6,593 (15.5%)	11,980 (7.0%)
Hispanic	766 (1.8%)	1,079 (2.5%)	1,482 (3.5%)	4,271 (10.0%)	7,598 (4.4%)
Non-Hispanic White	37,122 (86.7%)	36,744 (85.9%)	35,713 (83.7%)	28,660 (67.4%)	138,239 (80.9%)
Other	3,042 (7.1%)	2,323 (5.4%)	2,013 (4.7%)	1,916 (4.5%)	9,294 (5.4%)
Charlson comorbidity index					
0–1	32,898 (76.8%)	32,243 (75.4%)	31,788 (74.5%)	30,719 (72.2%)	127,648 (74.7%)
2	4,719 (11.0%)	4,546 (10.6%)	4,493 (10.5%)	4,397 (10.3%)	18,155 (10.6%)
(higher mortality risk) > 3	5,196 (12.1%)	5,968 (14.0%)	6,378 (15.0%)	7,433 (17.5%)	24,975 (14.6%)
Frailty index					
Q1 (0.02–0.10)	15,575 (36.4%)	14,665 (34.3%)	14,288 (33.5%)	13,885 (32.6%)	58,413 (34.2%)
Q2 (0.10–0.12)	7,751 (18.1%)	7,134 (16.7%)	6,663 (15.6%)	6,069 (14.3%)	27,617 (16.2%)
Q3 (0.12–0.15)	10,908 (25.5%)	10,738 (25.1%)	10,727 (25.1%)	10,674 (25.1%)	43,047 (25.2%)
(most frail) Q4 (0.15–0.49)	8,579 (20.0%)	10,220 (23.9%)	10,981 (25.7%)	11,921 (28.0%)	41,701 (24.4%)
Population characteristics					
Urban/rural					
Urban	38,868 (90.8%)	34,670 (81.1%)	29,579 (69.3%)	29,706 (69.8%)	132,823 (77.8%)
Rural	3,945 (9.2%)	8,087 (18.9%)	13,080 (30.7%)	12,843 (30.2%)	37,955 (22.2%)
Region					
Northeast	10,859 (25.4%)	7,894 (18.5%)	5,413 (12.7%)	4,836 (11.4%)	29,002 (17.0%)
Midwest	12,355 (28.9%)	10,819 (25.3%)	9,284 (21.8%)	5,426 (12.8%)	37,884 (22.2%)
South	12,279 (28.7%)	15,762 (36.9%)	18,636 (43.7%)	20,762 (48.8%)	67,439 (39.5%)
West	7,320 (17.1%)	8,282 (19.4%)	9,326 (21.9%)	11,525 (27.1%)	36,453 (21.3%)
# Ophthalmologists/100,000 population per HRR					
Q1 (2.0–5.2)	7,890 (18.4%)	9,531 (22.3%)	12,013 (28.2%)	12,250 (28.8%)	41,684 (24.4%)
Q2 (5.3–6.7)	9,996 (23.3%)	11,433 (26.7%)	11,258 (26.4%)	10,738 (25.2%)	43,425 (25.4%)
Q3 (6.8–8.7)	10,687 (25.0%)	10,905 (25.5%)	10,242 (24.0%)	10,924 (25.7%)	42,758 (25.0%)
Q4 (8.8–22.6)	14,240 (33.3%)	10,888 (25.5%)	9,146 (21.4%)	8,637 (20.3%)	42,911 (25.1%)

There were fewer beneficiaries diagnosed with cataracts residing in high SVI regions compared to low SVI regions (SVI Quartile 4 (Q4) 55.9% vs. Q1 67.1%, respectively, *p* < 0.001) (Table 2). Fewer beneficiaries in SVI Q4 received biometry (20.3% vs. 22.1%) and underwent cataract surgery (19.4% vs. 21.1%), compared to Q1 (*p* < 0.001 for all).

**Table 2:** Proportion of study cohort who received cataract diagnosis, biometry, and cataract surgery, stratified by SVI quartile

	SVI Quartile				Total	*P*-Value
	(lowest) Q1 (0.00–0.30) *N* = 42,813	Q2 (0.30–0.46) *N* = 42,757	Q3 (0.46–0.62) *N* = 42,659	(highest) Q4 (0.62–1.00) *N* = 42,549	170,778	
Cataract diagnosis	28,714 (67.1%)	27,239 (63.7%)	25,939 (60.8%)	23,793 (55.9%)	105,685	*p* < 0.001
Biometry	9,461 (22.1%)	9,457 (22.1%)	9,291 (21.8%)	8,618 (20.3%)	36,827	*p* < 0.001
Cataract surgery	9,054 (21.2%)	9,097 (21.3%)	8,956 (21.0%)	8,263 (19.4%)	35,370	*p* < 0.001

With increasing SVI quartile, fewer Black and Hispanic patients received a cataract diagnosis (Q4 47.2% and 48.5% vs. Q1 56.4% and 60.8%, respectively), had biometry (Q4 14.9% and 17.3%, vs. Q1 18.9% and 21.0%, respectively) and underwent cataract surgery (Q4 13.9% and 16.2% vs. Q1 18.6% and 19.8%, respectively). The p-trend across SVI quartiles within Black and Hispanic race/ethnicity ranged between < 0.001 and 0.003 for all cataract care events (eFigure 2a-c).

**Fig. 2:**
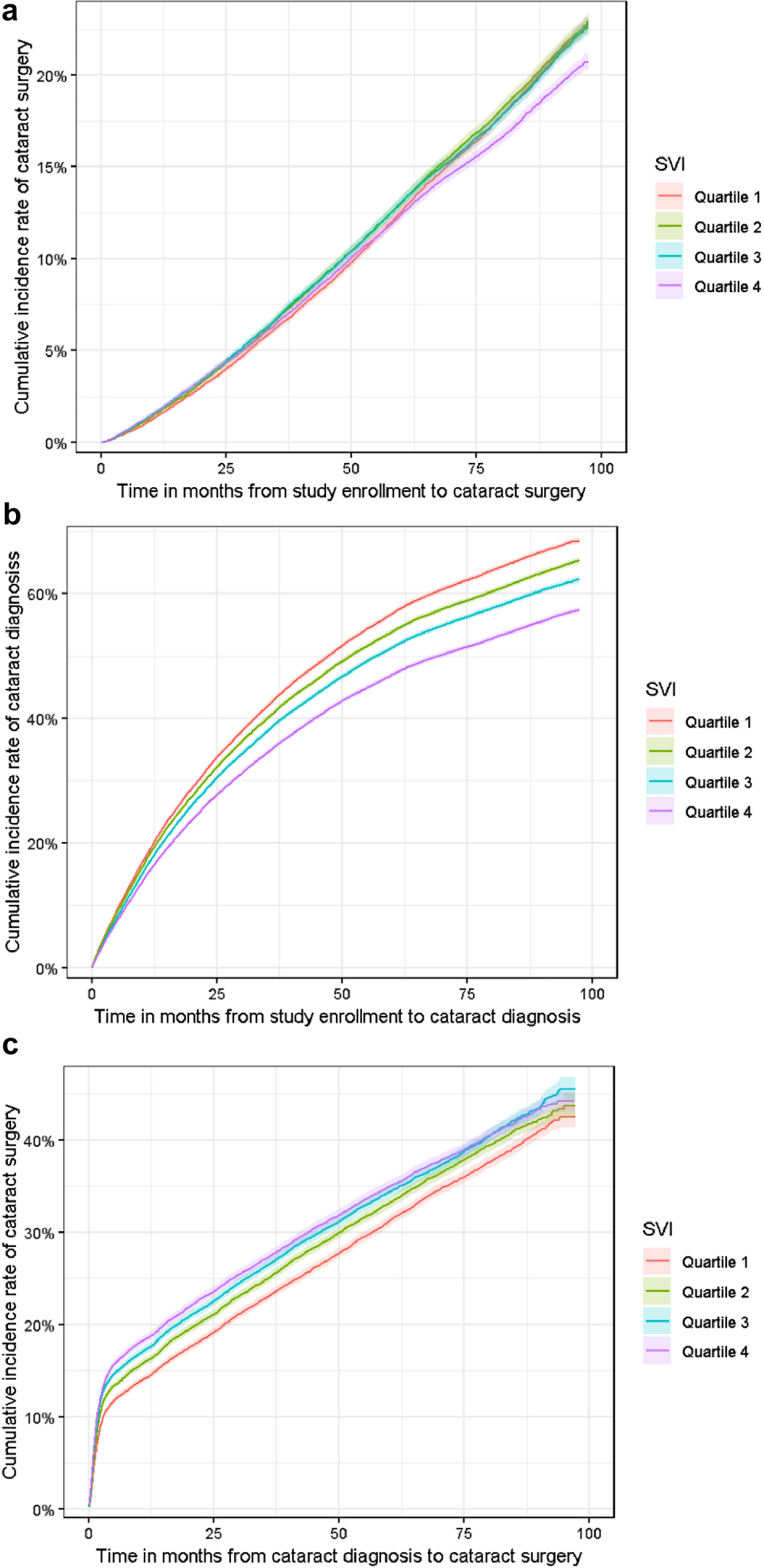
(**a**) Cumulative Incidence Curves Demonstrating Time Between Study Start and Receipt of Cataract Surgery, Stratified by SVI Quartile^a^. (**b**) Cumulative Incidence Curves Demonstrating Time Between Study Start and Cataract Diagnosis, Stratified by SVI Quartile^a^. (**c**) Cumulative Incidence Curves Demonstrating Time Between Cataract Diagnosis and Cataract Surgery, Stratified by SVI Quartile^b^. ^a^Cohort at risk is all Medicare patients enrolled at age 66 without diagnosis of cataract disease. ^b^Cohort at risk is the subset of Medicare patients age 66+ diagnosed with cataract disease

Patients in SVI Q4 appeared to wait longer for cataract surgery compared to patients in the other three quartiles (Fig. 2a). When we split the timeline into two distinct time periods, (1) study enrollment to cataract diagnosis, and (2) cataract diagnosis to cataract surgery, we found that patients in the higher SVI quartiles waited longer before receiving a diagnosis than patients in the lower SVI quartiles (Fig. 2b), but underwent cataract surgery sooner once diagnosed (Fig. 2c).

After adjusting for covariates, patients in higher SVI areas had lower rates of cataract diagnosis (Q2 HR: 0.92 95% CI (0.91, 0.94); Q3 0.86 (0.84, 0.87); Q4: 0.78 (0.77, 0.80), reference=Q1) and cataract surgery (Q3 HR: 0.95; 95% CI (0.92, 0.98) and Q4: 0.90 (0.87, 0.93), respectively, reference=Q1) (Table 3). Black and Hispanic patients had lower rates of diagnosis (HR: 0.74 (0.72, 0.76) and 0.82 (0.79, 0.85), respectively) and lower rates of surgery compared to White patients (HR: 0.65 (0.62, 0.69) and 0.85 (0.80, 0.90), respectively). Once a cataract diagnosis was made, we found that patients in higher SVI areas had higher rates of cataract surgery (Q2 HR: 1.04 (1.01, 1.07); Q3: 1.06 (1.03, 1.09); and Q4: 1.08 (1.05, 1.12), respectively, reference=Q1). Black patients had a lower rate of surgery compared to White patients (HR 0.80; (0.76, 0.84)). Of note, the models with biometry as the outcome were nearly identical to the surgery models, so the biometry model results are presented in the Appendix as eTable 4 rather than in Table 3.

**Table 3:** Association between SVI quartile and time to cataract care event

	Time to cataract diagnosis among all beneficiaries^a^	Time to cataract surgery among all beneficiaries^b^	Time to cataract surgery among beneficiaries with cataract diagnosis^c^
	Hazard Ratio (95% CI)	Hazard Ratio (95% CI)	Hazard Ratio (95% CI)
Age^d^	-	-	1.10 (1.09, 1.10)
Sex			
Male	Ref.	Ref.	Ref.
Female	1.48 (1.47, 1.50)	1.53 (1.49, 1.56)	1.17 (1.14, 1.19)
Race			
Non-Hispanic White	Ref.	Ref.	Ref.
Asian/Pacific Islander	1.00 (0.96, 1.04)	1.06 (0.98, 1.14)	1.07 (1.00, 1.15)
Black	0.74 (0.72, 0.76)	0.65 (0.62, 0.69)	0.80 (0.76, 0.84)
Hispanic	0.82 (0.79, 0.85)	0.85 (0.80, 0.90)	0.99 (0.93, 1.04)
Other	1.14 (1.11, 1.17)	0.98 (0.93, 1.02)	0.88 (0.84, 0.93)
Charlson comorbidity index			
0–1	Ref.	Ref.	Ref.
2	1.11 (1.09, 1.14)	1.08 (1.04, 1.12)	1.01 (0.97, 1.04)
(higher mortality risk) > 3	0.99 (0.97, 1.01)	1.01 (0.98, 1.04)	1.03 (1.00, 1.07)
Frailty index			
Q1 (0.02–0.10)	Ref.	Ref.	Ref.
Q2 (0.10–0.12)	1.11 (1.09, 1.14)	1.29 (1.25, 1.33)	1.05 (1.02, 1.08)
Q3 (0.12–0.15)	0.99 (0.97, 1.01)	1.39 (1.35, 1.43)	1.13 (1.10, 1.16)
(most frail) Q4 (0.15–0.49)	1.24 (1.22, 1.26)	1.41 (1.37, 1.46)	1.23 (1.19, 1.27)
Region			
Northeast	Ref.	Ref.	Ref.
Midwest	0.97 (0.95, 0.99)	1.21 (1.17, 1.26)	1.27 (1.22, 1.32)
South	1.07 (1.05, 1.09)	1.36 (1.31, 1.40)	1.33 (1.28, 1.37)
West	0.98 (0.96, 1.00)	1.14 (1.10, 1.18)	1.17 (1.13, 1.21)
Urban/rural			
Urban	Ref.	Ref.	Ref.
Rural	1.03 (1.01, 1.05)	1.06 (1.03, 1.09)	1.04 (1.02, 1.07)
# Ophthalmologists/100,000 population per HRR			
Q1 (2.0–5.2)	Ref.	Ref.	Ref.
Q2 (5.3–6.7)	0.98 (0.97, 1.00)	1.02 (0.99, 1.05)	1.03 (1.00, 1.06)
Q3 (6.8–8.7)	1.01 (0.99, 1.03)	0.95 (0.92, 0.98)	0.93 (0.91, 0.96)
Q4 (8.8–22.6)	1.00 (0.99, 1.02)	0.90 (0.88, 0.93)	0.89 (0.86, 0.92)
SVI quartile			
(lowest) 0.00–0.30	Ref.	Ref.	Ref.
0.30–0.46	0.92 (0.91, 0.94)	0.98 (0.95, 1.01)	1.04 (1.01, 1.07)
0.46–0.62	0.86 (0.84, 0.87)	0.95 (0.92, 0.98)	1.06 (1.03, 1.09)
(highest) 0.62–1.00	0.78 (0.77, 0.80)	0.90 (0.87, 0.93)	1.08 (1.05, 1.12)

^a^Cohort at risk is all Medicare patients enrolled at age 66 without diagnosis of cataract disease

^b^Cohort at risk is all Medicare patients enrolled at age 66 without diagnosis of cataract disease

^c^Cohort at risk is the subset of Medicare patients age 66 + diagnosed with cataract disease

^d^Age was not included as a covariate in the first two models because all patients were age 66 at the time of study enrollment

Of the 34,965 patients who underwent cataract surgery, 1.2% of patients had an ophthalmic complication within 30 days of surgery (eTable 5). Higher SVI did not appear to be associated with ophthalmic complications (eTable 6).

## Discussion

In our study of Medicare beneficiaries who did not have a prior cataract diagnosis, we found that patients in higher SVI quartiles had lower rates of diagnosis and surgery overall, but higher rates of cataract surgery once diagnosed. Furthermore, with increasing SVI quartile, Black and Hispanic patients had lower rates of diagnosis and surgery, but across all racial groups, once diagnosed, only Black patients had a lower rate of cataract surgery compared to White patients. Among patients who had cataract surgery, there was no association between SVI and ophthalmic complications.

Cataract disease is prevalent among older adults, increasing from 3.9% at age 55–64 years to 90% among 70 year olds [33] and 92.6% at age 80 years or older [34]. Previous studies have shown that high-deprivation neighborhoods are associated with a higher risk of age-related eye diseases, including cataract [35], and worse visual acuity before cataract surgery [32], and one study found that higher SVI areas were associated with lower cataract surgery utilization [36]. Similarly, our study adds further evidence that patients in higher SVI quartiles have lower rates of cataract diagnosis and cataract surgery overall.

However, higher SVI was also associated with higher rates of cataract surgery once patients had been diagnosed, a finding that merits careful consideration of several potential mechanisms. First, high-SVI patients may present with more severe disease at diagnosis [32], creating greater urgency of care and prioritization of surgical intervention. For example, there may be greater prioritization of more symptomatic patients or threshold effects in referral and surgery uptake. Provider-level adaptation may also play a role, whereby clinicians caring for high-SVI populations may adopt lower thresholds for surgical referral in response to anticipated barriers during follow-up or postoperative care.

Correspondingly, our study suggests that low-SVI patients diagnosed earlier in the disease course may have a longer pre-surgical observation window that inflates apparent time-to-surgery relative to high-SVI patients. Others have shown that physicians practicing in densely populated urban areas with greater affluence and resources tend to diagnose patients earlier in their disease course [37]. Our study supports this phenomenon, as patients who lived in areas with more ophthalmologists per capita appeared to have lower rates of cataract surgery than those in areas with fewer ophthalmologists. This finding is also consistent with prior literature that shows that cataract patients do not necessarily proceed with surgery until their vision is affecting their QoL [38–40]. However, our findings may be confounded by overlapping constructs that warrant further investigation, such as differences in the timing of recorded diagnosis in the course of the underlying disease, variations in urban vs. rural practice patterns, greater patient selectivity and ascertainment bias, regional coding differences or other unmeasured system-level factors [37]. 

Regardless of SVI quartile, when looking at our entire Medicare study cohort without cataract disease at age 66, Black and Hispanic patients had a lower rate of cataract diagnosis and cataract surgery than White patients. This adds to the literature that shows that racial minority groups experience greater challenges to receiving timely cataract surgery compared with White patients [8]. For example, Black patients are less likely to see an ophthalmologist or optometrist for eye care than White patients [41, 42], despite higher prevalence of cataract in the Black population [43]. In addition, although Hispanic individuals have a greater prevalence of visually significant cataract, they are less likely to receive surgery due to language and financial barriers [7]. Moreover, our study suggests that for Black patients, these disparities persist even after diagnosis, as Black patients who went on to be diagnosed with cataract disease had lower rates of cataract surgery compared to White patients. Our findings conflict with a prior study that found that Black patients have higher rates of cataract surgery once diagnosed [42], though this study did not account for the impact of SVI on timing of cataract care.

Prior studies have indicated some association between social vulnerability and surgical outcomes, such as visual acuity [36]. Patients in higher SVI may also be seen less frequently in the postoperative period due to limited access to care, and thus fewer complications may be documented in the medical record [44]. Although our study detected a low incidence of complications after cataract surgery overall, it was likely underpowered to detect a clinically meaningful difference in the complication rate between SVI quartiles. However, the overall rarity of complications reflects the fact that cataract surgery is highly standardized, and our findings are consistent with other studies that document a low risk of complications or readmissions after cataract surgery [34, 45]. 

Delayed cataract surgery can lead to motor vehicle accidents, depression, frailty, vision loss, reduced QoL, and increased fall rates during the waiting period [5, 10–14, 38]. Notably, our study found that patients with a higher frailty index had higher rates of undergoing cataract surgery, suggesting that frail patients and their clinicians may be more motivated to pursue timely intervention to minimize risks of delayed cataract surgery [1, 46]. Given the importance of vision for preserving functional independence and quality of life, early diagnosis and discussion of the risks and benefits of surgical intervention are essential for optimizing outcomes in frail older adults.

Collectively, our study highlights the need to improve access to ophthalmologists for timely diagnosis of cataracts. Although our findings suggest that for most patients, disparities do not accumulate at each stage of cataract care and can mostly explained by the timing of cataract diagnosis, there may still be other factors that continue to exacerbate cumulative disparities in receipt of cataract surgery, especially for Black Medicare enrollees. Additionally, because all of our patients were Medicare beneficiaries, the lack of insurance is not a factor in our study with regards to care access. Instead, these disparities may exist across SVI quartiles due to physician-patient factors, socioeconomic status, and educational levels [47]. For instance, our study demonstrated that females had higher rates of undergoing cataract surgery, which may be partly explained by previously described sex differences in healthcare-seeking behavior [48]. Future interventions should work toward greater accessibility to cataract screening and patient-tailored education in high-SVI communities.

There are several limitations due to the study’s retrospective design. First, to have a 12-month look back for risk stratification, patients aged < 66 were excluded from the study. It is possible that many patients who had cataract disease received their surgery upon Medicare enrollment at 65, which would not be reflected in our study cohort. Second, the first recorded diagnosis of cataract disease may not necessarily represent the true onset of clinically significant cataract or the actual timing of the first ophthalmic evaluation. Other factors including delayed coding, reduced eye-care utilization, lower symptom burden reaching documentation threshold, and differences in referral pathways can affect the timing of a new cataract diagnosis in claims data. In addition, linking census tracts to beneficiary zip codes may lead to non-differential misclassification bias in assigning SVI, which would attenuate the observed effect of SVI on cataract care towards the null hypothesis. Others have shown that SVI has robust predictive power to predict health outcomes when measured at the census tract level. Therefore, the true SVI-care associations may be larger than those reported here [49]. The race category in Medicare claims data may not accurately reflect the true racial identity of the study cohort due to the limited availability of race/ethnicity categories during Medicare enrollment. Furthermore, cataract care events may exclude care events in patients who were diagnosed with cataract disease towards the end of 2021 due to insufficient follow-up after diagnosis. Our study also did not assess visual acuity outcomes, reoperations, readmissions, or other complications, and we did not have access to clinical variables that are not readily available in claims data. The lack of variables representing disease progression in our analyses is particularly important due to the potential for more severe disease at presentation to expedite the time from diagnosis to surgery. Transition to Medicare Advantage during the study period (2014–2021) may represent a source of informative censoring, as high SVI patients may have disenrolled from fee-for-service Medicare at differential rates, potentially biasing time-to-event estimates. Despite these limitations, this study advances our understanding of the mechanisms by which disparities in cataract care might accumulate [36]. 

## Conclusion

In conclusion, this retrospective cohort study suggests that higher SVI is associated with disparities in receipt of cataract surgery among Medicare beneficiaries, which may be mediated by delays in cataract diagnosis. Although our findings suggest that disparities may largely reflect delays in diagnosis rather than accruing independently at each stage of care, other factors may contribute to cumulative disparities, including race and access to ophthalmological care. Further investigation of SDOH factors underlying disparities at each stage of cataract care is needed to identify and target interventions at each of these critical time points. Future directions include closer examination of sociocultural factors influencing cataract care in minority populations, as well as the evaluation of whether proposed interventions, such as enhanced screening or educational programs, can meaningfully reduce gaps in cataract diagnosis and treatment. Ultimately, barriers to equitable care must be addressed to ensure timely diagnosis of cataract disease and access to surgery among under-resourced populations.

## Supplementary Information

Below is the link to the electronic supplementary material.

Supplementary Material 1

## Data Availability

CLC and SG had full access to the data and are responsible for the data analysis. This data is not available for data sharing according to the Center for Medicare and Medicaid Services data use agreement signed by the study PI.
