# Supplementary material for: Association between social vulnerability index and cataract surgery care in medicare beneficiaries: a retrospective cohort study
Source: BMC Ophthalmol. 2026 Jul 9;26:402. doi: 10.1186/s12886-026-05110-2 (PMC13348854; doi:10.1186/s12886-026-05110-2)
Supplement: Supplementary file 1 — Supplementary Material 1 [file 12886_2026_5110_MOESM1_ESM.docx]

**Association between Social Vulnerability Index and Cataract Surgery Care in Medicare Beneficiaries**

**Online Supplement**

This supplementary material has been provided by the authors to give readers additional information about their work.

**Table of Contents**

**eTable 1.** ICD-9 and ICD-10 Codes Used to Define Cataract Diagnosis

**eTable 2.** ICD-9 and ICD-10 Codes Used to Define Ophthalmic Complications

**eTable 3.** Assessment of Collinearity Among Variables Included in the Models

**eTable 4.**  Association between SVI Quartile and Time to Biometry

**eTable 5.** Ophthalmic Complications within 30 Days, Stratified by SVI Quartile

**eTable 6.**  Association between SVI Quartile and Ophthalmic Complications Among Medicare Beneficiaries Who Had Cataract Surgery

**eFigure 1.** CONSORT Diagram

**eFigure 2a.** Proportion of Study Cohort Who Received Cataract Diagnosis, Stratified by SVI and Race

**eFigure 2b.** Proportion of Study Cohort Who Received Biometry, Stratified by SVI and Race

**eFigure 2c.** Proportion of Study Cohort Who Received Cataract Surgery, Stratified by SVI and Race

**eTable 1. ICD-9 and ICD-10 Codes Used to Define Cataract Diagnosis**

| ICD9 | ICD10 |
| --- | --- |
| '366.01', '366.02', '366.03', '366.04', '366.09', '366.10', '366.12', '366.13', '366.14', '366.15', '366.16', '366.17', '366.18', '366.19', '366.20', '366.21', '366.22', '366.23', '366.30', '366.32', '366.33', '366.34', '366.41', '366.42', '366.43', '366.44', '366.45', '366..46', '36650', '366.51', '366.52', '366.53', '366.8', '366.9', '379.26', '379.31', '379.39', '743.31', '743.32', '743.33', '996.53', 'V43.1' | 'E08.36', 'E09.36', 'E10.36', 'E11.36', 'E13.36',  'H25.011', 'H25.012', 'H25.013', 'H25.019', 'H25.031', 'H25.032', 'H25.033', 'H25.039', 'H25.041', 'H25.042', 'H25.043', 'H25.049', 'H25.091', 'H25.092', 'H25.093',  'H25.099', 'H25.10',  'H25.11',  'H25.12',  'H25.13',  'H25.20',  'H25.21',  'H25.22',  'H25.23',  'H25.811', 'H25.812', 'H25.813', 'H25.819', 'H25.89', 'H25.9', 'H26.001', 'H26.002', 'H26.003', 'H26.009', 'H26.011', ‘H26.012', 'H26.013', 'H26.019', 'H26.031', 'H26.032', 'H26.033', 'H26.039', 'H26.041', 'H26.042', 'H26.043', 'H26.049', 'H26.051', 'H26.052', 'H26.053', 'H26.059', 'H26.061', 'H26.062', 'H26.063', 'H26.069', 'H26.09', 'H26101', 'H26102', 'H26.103', 'H26.109', 'H26.111', 'H26.112', 'H26.113', 'H26.119', 'H26.121', 'H26.122', 'H26.123', 'H26.129', 'H26.131', 'H26.132', 'H26.133', 'H26.139', 'H26.20',  'H26.211', 'H26.212', 'H26.213', 'H26.219', 'H26.221', 'H26.222', 'H26.223', 'H26.229', 'H26.30', 'H26.31', 'H26.32', 'H26.33', 'H26.40', 'H26.411',  'H26.412', 'H26.413', 'H26.419', 'H26.491', 'H26.492', 'H26.493', 'H26.499', 'H26.8',  'H26.9', 'Q12.0' |

**eTable 2. ICD-9 and ICD-10 Codes Used to Define Ophthalmic Complications**

| Ophthalmic Complications | ICD-10 Complication Code | ICD-9 Description | ICD-9 Complication Code |
| --- | --- | --- | --- |
| Keratopathy (bullous aphakic) | H59.01* | Bullous keratopathy | 371.23 |
| Cataract (lens) fragment in eye | H59.02* | Cataract fragments in eye following cataract surgery | 998.82 |
| Cystoid macular edema | H59.03* | Cystoid macular degeneration | 362.53 |
| Other disorders of eye | H59.09* | - | - |
| Intraoperative or postprocedural hemorrhage of eye | H59.11*, H59.31* | Hemorrhage complicating a procedure | 998.11 |
| Accidental puncture and laceration of eye | H59.21* | Accidental puncture or laceration during a procedure, not elsewhere classified | 998.2 |
| Postprocedural hematoma of eye | H59.33* | Hematoma complicating a procedure | 998.12 |
| Postprocedural seroma or eye | H59.35* | Seroma complicating a procedure | 998.13 |
| Other intraoperative or postprocedural complications of eye, NOS | H59.88,  H59.89 | Complications affecting other specified body systems, not elsewhere classified | 997.99 |
| Mechanical complication of intraocular lens | T85.21*, T85.22*, T85.29* | Mechanical complication due to ocular lens prosthesis | 996.53 |
| Retinal detachment | H33.0*, H33.4* | Retinal detachments and defects | 361.81, 361.0x |

**eTable 3. Assessment of Collinearity Among Variables Included in the Models**

|  | **Variance Inflation Factors (VIF)** | |
| --- | --- | --- |
|  | **Model A1-Time to cataract diagnosis among all beneficiaries**  **and**  **Model B2-Time to cataract surgery among all beneficiaries** | **Model C3-Time to cataract surgery among beneficiaries with cataract diagnosis** |
| **Variable** | VIF | VIF |
| **Age** | - | 1.01 |
| **Sex: Female** | 1.03 | 1.03 |
| **Race** |  |  |
| **Asian/Pacific Islander** | 1.03 | 1.03 |
| **Black** | 1.09 | 1.07 |
| **Hispanic** | 1.06 | 1.04 |
| **Other** | 1.02 | 1.02 |
| **Charlson comorbidity index** |  |  |
| **2** | 1.09 | 1.08 |
| **(higher mortality risk) >3** | 1.31 | 1.26 |
| **Frailty index** |  |  |
| **Q2 (0.10 - 0.12)** | 1.25 | 1.31 |
| **Q3 (0.12 - 0.15)** | 1.36 | 1.44 |
| **(most frail) Q4 (0.15 - 0.49)** | 1.69 | 1.69 |
| **Region** |  |  |
| **Midwest** | 1.98 | 1.95 |
| **South** | 2.29 | 2.25 |
| **West** | 1.91 | 1.86 |
| **Urban/rural: Rural** | 1.11 | 1.12 |
| **# Ophthalmologists/100000 population per HRR** |  |  |
| **Q2 (5.3 – 6.7)** | 1.57 | 1.57 |
| **Q3 (6.8 – 8.7)** | 1.59 | 1.58 |
| **Q4 (8.8 – 22.6)** | 1.73 | 1.73 |
| **SVI quartile** |  |  |
| **0.30 - 0.46** | 1.53 | 1.46 |
| **0.46 - 0.62** | 1.61 | 1.53 |
| **(highest) 0.62 - 1.00** | 1.76 | 1.63 |

**eTable 4.** **Association between SVI Quartile and Time to Biometry**

|  | Time from study enrollment to biometry among all beneficiaries | Time from cataract diagnosis to biometry among beneficiaries with a cataract diagnosis |
| --- | --- | --- |
|  | **Hazard Ratio (95% CI)** | **Hazard Ratio (95% CI)** |
| Age^a^ |  | 1.10 (1.10, 1.11) |
| Sex |  |  |
| Male | Ref. | Ref. |
| Female | 1.54 (1.51, 1.58) | 1.18 (1.16, 1.21) |
| Race |  |  |
| Non-Hispanic White | Ref. | Ref. |
| Asian/Pacific Islander | 1.08 (1.01, 1.16) | 1.10 (1.03, 1.18) |
| Black | 0.67 (0.64, 0.71) | 0.82 (0.78, 0.86) |
| Hispanic | 0.87 (0.82, 0.91) | 1.00 (0.95, 1.06) |
| Other | 0.98 (0.93, 1.03) | 0.89 (0.85, 0.93) |
| Charlson comorbidity index |  |  |
| 0-1 | Ref. | Ref. |
| 2 | 1.08 (1.05, 1.12) | 1.01 (0.98, 1.04) |
| (higher mortality risk) >3 | 1.01 (0.97, 1.04) | 1.03 (1.00, 1.07) |
| Frailty index |  |  |
| Q1 (0.02 - 0.10) | Ref. | Ref. |
| Q2 (0.10 - 0.12) | 1.29 (1.25, 1.33) | 1.05 (1.02, 1.08) |
| Q3 (0.12 - 0.15) | 1.40 (1.36, 1.44) | 1.14 (1.11, 1.17) |
| (most frail) Q4 (0.15 - 0.49) | 1.41 (1.37, 1.46) | 1.23 (1.19, 1.27) |
| Region |  |  |
| Northeast | Ref. | Ref. |
| Midwest | 1.17 (1.13, 1.22) | 1.22 (1.18, 1.27) |
| South | 1.31 (1.27, 1.35) | 1.28 (1.24, 1.32) |
| West | 1.10 (1.07, 1.15) | 1.13 (1.09, 1.17) |
| Urban/rural |  |  |
| Urban | Ref. | Ref. |
| Rural | 1.05 (1.02, 1.07) | 1.02 (1.00, 1.05) |
| # Ophthalmologists/100000 population per HRR |  |  |
| Q1 (2.0 – 5.2) | Ref. | Ref. |
| Q2 (5.3 – 6.7) | 1.01 (0.98, 1.04) | 1.03 (1.00, 1.06) |
| Q3 (6.8 – 8.7) | 0.95 (0.93, 0.98) | 0.94 (0.91, 0.97) |
| Q4 (8.8 – 22.6) | 0.92 (0.90, 0.95) | 0.91 (0.89, 0.94) |
| SVI quartile |  |  |
| (lowest) 0.00 - 0.30 | Ref. | Ref. |
| 0.30 - 0.46 | 0.98 (0.95, 1.01) | 1.04 (1.01, 1.07) |
| 0.46 - 0.62 | 0.95 (0.92, 0.98) | 1.06 (1.03, 1.09) |
| (highest) 0.62 - 1.00 | 0.91 (0.88, 0.93) | 1.09 (1.05, 1.12) |

^a^Age was not included as a covariate in the first model among all beneficiaries because all patients were age 66 at the time of study enrollment.

**eTable 5. Ophthalmic Complications within 30 Days, Stratified by SVI Quartile^a^**

|  | **SVI** | | | | |
| --- | --- | --- | --- | --- | --- |
|  | **Q1**  **(0.00 - 0.30)** | **Q2**  **(0.30 - 0.46)** | **Q3**  **(0.46 - 0.62)** | **Q4**  **(0.62 - 1.00)** | **Total** |
|  | N=8,935 | N=9,000 | N=8,857 | N=8,173 | N=34,965 |
| Any complication | 115 (1.3%) | 118 (1.3%) | 95 (1.1%) | 98 (1.2%) | 426 (1.2%) |
| Keratopathy (bullous aphakic) | 0 (0.0%) | <11 (0.0%) | 0 (0.0%) | 0 (0.0%) | <11 (0.0%) |
| Cataract (lens) fragment in eye | 39 (0.4%) | 36 (0.4%) | 31 (0.4%) | 41 (0.5%) | 147 (0.4%) |
| Cystoid macular edema | 19 (0.2%) | 19 (0.2%) | 14 (0.2%) | 15 (0.2%) | 67 (0.2%) |
| Other disorders of eye | <11 (0.0%) | <11 (0.0%) | <11 (0.0%) | <11 (0.0%) | 12 (0.0%) |
| Intraoperative or postprocedural hemorrhage of eye | <11 (0.0%) | 0 (0.0%) | <11 (0.0%) | 0 (0.0%) | <11 (0.0%) |
| Accidental puncture and laceration of eye | <11 (0.0%) | 0 (0.0%) | <11 (0.0%) | <11 (0.0%) | <11 (0.0%) |
| Postprocedural hematoma of eye | 0 (0.0%) | 0 (0.0%) | 0 (0.0%) | 0 (0.0%) | 0 (0.0%) |
| Postprocedural seroma of eye | <11 (0.0%) | 0 (0.0%) | <11 (0.0%) | 0 (0.0%) | <11 (0.0%) |
| Other intraoperative or postprocedural complications of eye, NOS | <11 (0.0%) | <11 (0.0%) | 0 (0.0%) | <11 (0.0%) | <11 (0.0%) |
| Mechanical complication of intraocular lens | 28 (0.3%) | 26 (0.3%) | 17 (0.2%) | 27 (0.3%) | 98 (0.3%) |
| Retinal detachment | 28 (0.3%) | 36 (0.4%) | 32 (0.4%) | 17 (0.2%) | 113 (0.3%) |

1. Results for cell size <11 not presented to comply with CMS cell size suppression policy. (https://resdac.org/articles/cms-cell-size-suppression-policy)

**eTable 6. Association between SVI Quartile and Ophthalmic Complications Among Medicare Beneficiaries Who Had Cataract Surgery**

|  | **Odds ratio** |
| --- | --- |
| **Age at surgery** | 0.95 (0.90, 0.99) |
| **Sex** |  |
| Male | Ref. |
| Female | 0.64 (0.52, 0.78) |
| **Race** |  |
| Non-Hispanic White | Ref. |
| Asian/Pacific Islander | 1.09 (0.59, 1.99) |
| Black | 0.82 (0.51, 1.33) |
| Hispanic | 1.22 (0.76, 1.96) |
| Other | 0.75 (0.46, 1.23) |
| **Charlson comorbidity index** |  |
| 0-1 | Ref. |
| 2 | 1.02 (0.74, 1.40) |
| >3 (higher mortality risk) | 1.14 (0.85, 1.53) |
| **Frailty index** |  |
| Q1 (0.02 - 0.10) | Ref. |
| Q2 (0.10 - 0.12) | 0.93 (0.68, 1.27) |
| Q3 (0.12 - 0.15) | 0.91 (0.69, 1.19) |
| Q4 (0.15 - 0.49) (most frail) | 1.05 (0.78, 1.40) |
| **Region** |  |
| Northeast | Ref. |
| Midwest | 0.89 (0.62, 1.28) |
| South | 1.06 (0.77, 1.47) |
| West | 1.05 (0.73, 1.49) |
| **Urban/rural** |  |
| Urban | Ref. |
| Rural | 0.79 (0.60, 1.05) |
| **# Ophthalmologists/100000 population per HRR** |  |
| Q1 (2.0 – 5.2) | Ref. |
| Q2 (5.3 – 6.7) | 1.13 (0.84, 1.52) |
| Q3 (6.8 – 8.7) | 0.95 (0.70, 1.30) |
| Q4 (8.8 – 22.6) | 1.13 (0.82, 1.54) |
| **Annual surgical volume** |  |
| Q1 (1-37) | Ref. |
| Q2 (38-65) | 0.78 (0.61, 1.02) |
| Q3 (66-117) | 0.58 (0.43, 0.77) |
| Q4 (118-1119) | 0.48 (0.35, 0.66) |
| **SVI quartile** |  |
| 0.00 - 0.30 (lowest) | Ref. |
| 0.30 - 0.46 | 1.07 (0.82, 1.41) |
| 0.46 - 0.62 | 0.89 (0.66, 1.19) |
| 0.62 - 1.00 (highest) | 0.96 (0.71, 1.31) |

**eFigure 1.** CONSORT Diagram


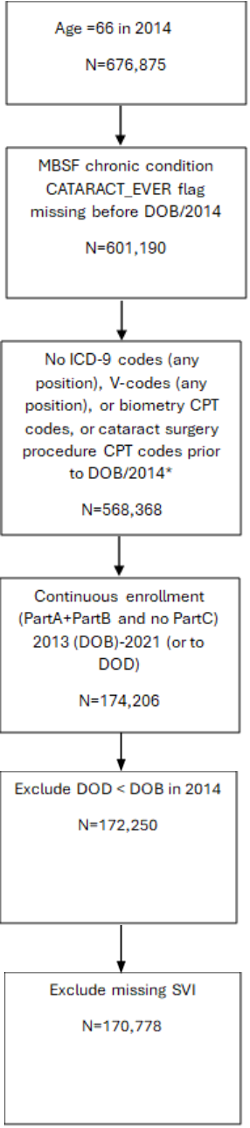


**eFigure 2a.** Proportion of Study Cohort Who Received Cataract Diagnosis, Stratified by SVI and Race^a^


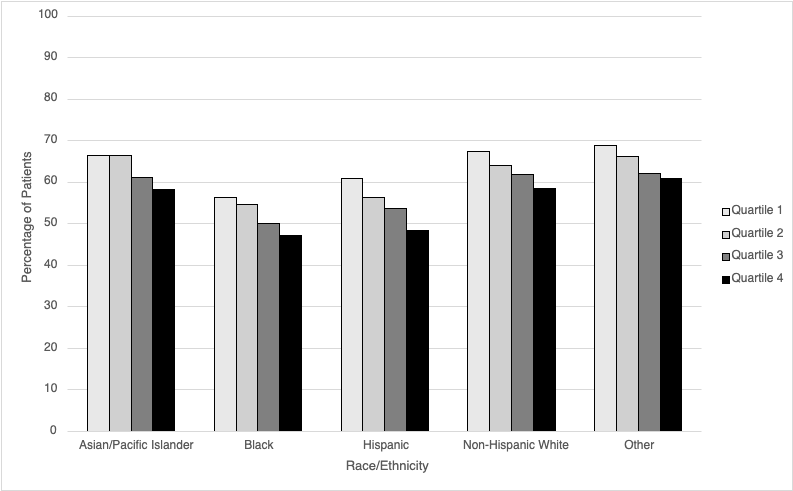


1. The p-value for trend across SVI quartiles was <0.001 for each respective category of race/ethnicity.

**eFigure 2b.** Proportion of Study Cohort Who Received Biometry, Stratified by SVI and Race^a^


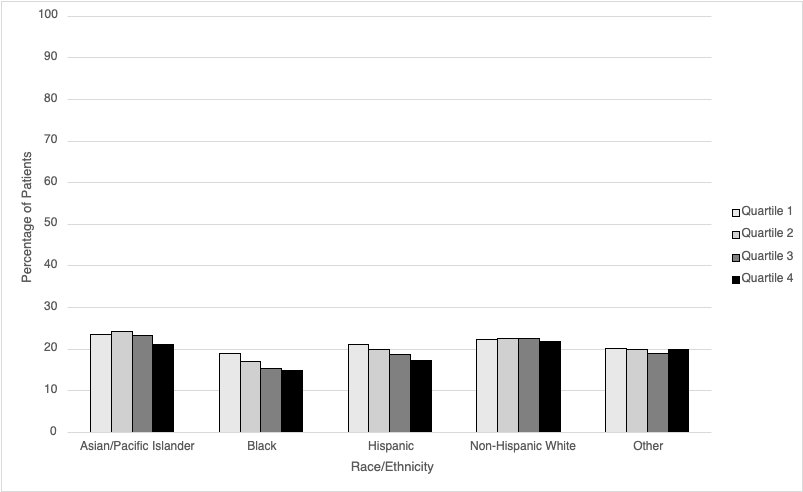


1. The p-value for trend across SVI quartiles was <0.001 for Black patients and 0.003 for Hispanic patients. The p-value for trend was not statistically significant for the other categories of race/ethnicity.

**eFigure 2c.** Proportion of Study Cohort Who Received Cataract Surgery, Stratified by SVI and Race^a^


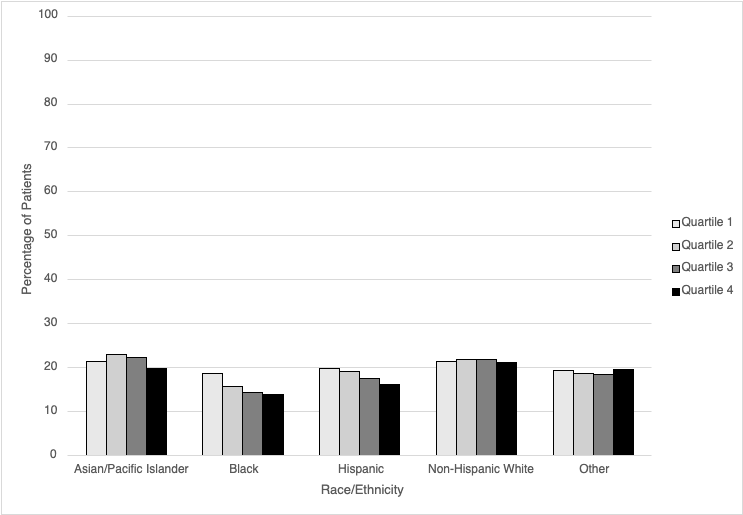


1. The p-value for trend across SVI quartiles was <0.001 for Black patients and 0.002 for Hispanic patients. The p-value for trend was not statistically significant for the other categories of race/ethnicity.
